# The mitotic functions of a fission yeast CK1 enzyme are regulated by Cdk1-dependent and auto-phosphorylation

**DOI:** 10.1016/j.jbc.2025.111007

**Published:** 2025-12-05

**Authors:** Kazutoshi Akizuki, Sierra N. Cullati, Alyssa E. Johnson, Jun-Song Chen, Alaina H. Willet, Kathleen L. Gould

**Affiliations:** Department of Cell and Developmental Biology, Vanderbilt University School of Medicine, Nashville, Tennessee, USA

**Keywords:** casein kinase 1, hhp2, cdk1, phosphoregulation, mitotic checkpoint, cell cycle control, *Schizosaccharomyces pombe*

## Abstract

CK1 enzymes are conserved regulators of diverse cellular processes. In *Schizosaccharomyces pombe*, the CK1 orthologs of CK1δ and CK1ε, Hhp1 and Hhp2, are required for a mitotic checkpoint that delays cytokinesis when the mitotic spindle is disrupted. Here, we show that Hhp2, but not Hhp1, undergoes transient hyperphosphorylation during mitosis. Hhp2 autophosphorylates at four residues and is phosphorylated by the cyclin-dependent kinase Cdk1 at three additional sites. Functionally, these phosphorylation events inhibit Hhp2 catalytic activity, as phospho-ablating mutants exhibited enhanced *in vitro* kinase activity. *In vivo*, a mutant combining all seven sites (*hhp2-7A*) behaved as a gain-of-function mutant in the mitotic checkpoint and also had the unexpected phenotype of accelerating mitosis and cytokinesis in unperturbed conditions. Further genetic analyses indicated that Hhp2 likely promotes mitotic progression in parallel with the Polo-like kinase, Plo1. These findings establish that mitotic phosphorylation of Hhp2 serves as a negative regulatory mechanism that silences checkpoint activity and modulates cell cycle timing. Because mitotic phosphorylation of human CK1δ has been observed, our results suggest that Cdk1-mediated inhibition of CK1 enzymes is a conserved mechanism coupling the core cell cycle control machinery to CK1-dependent signaling pathways.

CK1 enzymes are conserved, eukaryotic serine/threonine kinases that have been implicated in normal cellular processes such as DNA repair, circadian rhythm, and Wnt signaling, and in diseases such as cancer, neurodegenerative diseases, and sleep disorders (1, 2, 3, 4, 5). The human CK1 kinase family consists of seven paralogs, CK1α, α-like (α2), γ1–3, δ, and ε. CK1α/α-like and CK1δ/ε are soluble enzymes that localize ubiquitously in the cell, whereas CK1γ1–3 are palmitoylated and compartmentalized on membranes (5, 6). Among soluble CK1 enzymes, CK1δ/ε are highly related to each other (3) and to soluble CK1s in other organisms, such as Hhp1 and Hhp2 in the fission yeast *Schizosaccharomyces pombe* and Hrr25 in *Saccharomyces cerevisiae* (7, 8).

All CK1 enzymes are monomers with conserved N-terminal catalytic domains and C-termini of different lengths and sequences (3). Multiple studies have demonstrated that the C-termini of CK1δ/ε and of the related yeast enzymes are substrates for autophosphorylation *in vitro* and *in vivo* (8, 9, 10, 11, 12, 13, 14, 15). The C-termini of these enzymes can also be phosphorylated by other kinases such as PKA and Chk1 (16, 17). The phosphorylated C-termini act as pseudosubstrates and compete with substrates for access to the catalytic cleft, thus inhibiting kinase activity (2, 10, 12, 15). Two positively charged binding pockets (Site 1 and Site 2) have been shown to specifically interact with phosphorylated substrates and the phosphorylated C-terminus (12, 18, 19, 20). Site 1 is unique to the CK1 family and lies in the kinase C lobe adjacent to the active site, while Site 2 wraps around behind the substrate binding groove and overlaps with the HRD motif found in many kinase families (21, 22). Mutation of the basic residues in Site 1 disrupts binding to the phosphorylated C-terminus and also prevents substrate phosphorylation in *S*. *pombe* Hhp1 and human CK1ε (12). Mutation of Site 2 affects the conformation of the activation loop and shape of the substrate binding groove in CK1δ (18). When CK1 interacts with multiply phosphorylated substrates, both Site 1 and Site 2 coordinate phosphate groups (18, 19, 20).

Although phosphorylation of CK1 C-termini is easily detected *in vitro*, the basal amount of C-terminal autophosphorylation in mammalian cells appears to be low. Specifically, when cell and tissue samples are examined by immunoblotting, reduced SDS-PAGE mobility forms that correspond to autophosphorylation are seldom observed, suggesting that in cells, these CK1 enzymes are hypophosphorylated most of the time (15, 23, 24, 25, 26, 27, 28). Treating cells with phosphatase inhibitors such as calyculin A or okadaic acid increases detection of slower mobility phosphorylated forms of CK1δ/ε, and cellular stress conditions such as heat shock and replication stress also increase detection of phosphorylated Hhp1 (12, 15, 24, 26, 27). These observations indicate that phosphorylation of the CK1δ/ε subfamily does occur *in vivo*, but the enzymes are rapidly dephosphorylated by cellular phosphatases in a “futile phosphorylation/dephosphorylation cycle” (26, 29, 30), which led to the idea that under basal conditions, CK1δ/ε subfamily kinases are constitutively active (2, 26). Despite the idea that they are already dephosphorylated and active, stimuli such as a metabotropic glutamate receptor agonist, activation of Wnt signaling, and inhibition of PKA enhance CK1δ and/or CK1ε activity (17, 30, 31, 32). Clearly, endogenous CK1δ/ε subfamily enzymes are dynamically phosphorylated and dephosphorylated *in vivo*, but the physiological significance of when and why this happens is uncertain.

In a previous study, we noticed that endogenous human CK1δ appeared to be hyperphosphorylated during mitosis (33). Subsequently, mitotic hyperphosphorylation of overexpressed CK1δ was also reported (34). In the present study, we investigated whether the CK1δ/ε-related Hhp1 and/or Hhp2 are inhibited *via* C-terminal phosphorylation during mitosis in *S*. *pombe*, a model organism particularly well-suited for cell cycle studies (35) and in which mitotic functions of Hhp1/2 have been characterized (36, 37, 38). Hhp1 is far more abundant than Hhp2, and therefore Hhp2’s roles can be masked by the presence of Hhp1 (39). For example, unlike the deletion of *hhp1* alone, the deletion of *hhp2* alone does not cause a noticeable morphological or growth defect (7, 39, 40). However, the dramatic defects of double *hhp1 hhp2* mutants compared to the single *hhp1* mutant demonstrate that Hhp1 and Hhp2 function redundantly in meiosis (41, 42, 43), mitotic commitment (37, 38), and in a mitotic checkpoint (36, 39).

In the mitotic checkpoint, Hhp1 and Hhp2 act upstream of Dma1, a ubiquitin ligase that inhibits the Hippo-related septation initiation network (SIN) (44, 45, 46, 47, 48, 49). Components of the SIN assemble and signal from the spindle pole body (SPB) to trigger cytokinesis (46, 50). The most upstream activator of SIN signaling at the SPB is Plo1 (51, 52, 53). Hhp1 and Hhp2 inhibit SIN signaling by phosphorylating the SIN SPB scaffold protein Sid4 at T275 and S278, creating a docking site for the FHA domain of Dma1 (36). Dma1 then ubiquitinates Sid4 to antagonize Plo1 SPB localization, thus inhibiting the SIN during the mitotic checkpoint (54). Mutating Sid4 residue T275 to alanine or inhibiting Hhp1/2 kinase activity to abolish Sid4 phosphorylation bypasses the mitotic checkpoint delay (36). Thus, Hhp1 and Hhp2-dependent Sid4 phosphorylation is the most upstream event identified that evokes the Dma1-dependent mitotic checkpoint (47).

We report here that like CK1δ, Hhp2, but not Hhp1, is transiently hyperphosphorylated on its C-terminus during mitosis. Interestingly, we found that Cdk1 and autophosphorylation are both responsible for the mitotic phosphorylation and that these phosphorylation events both inhibit kinase activity *in vitro*. By making a phosphoablating mutant that combines all of the sites, we determined that mitotic phosphorylation inhibits Hhp2 function in the checkpoint. Surprisingly, we also found that the phosphoablating *hhp2* mutant advanced mitotic progression when cells were not experiencing a prolonged checkpoint arrest, unmasking a previously unrecognized Hhp2 role in cell cycle control.

## Results

### Hhp2 is hyperphosphorylated and inhibited during mitosis

**Figure 1:**
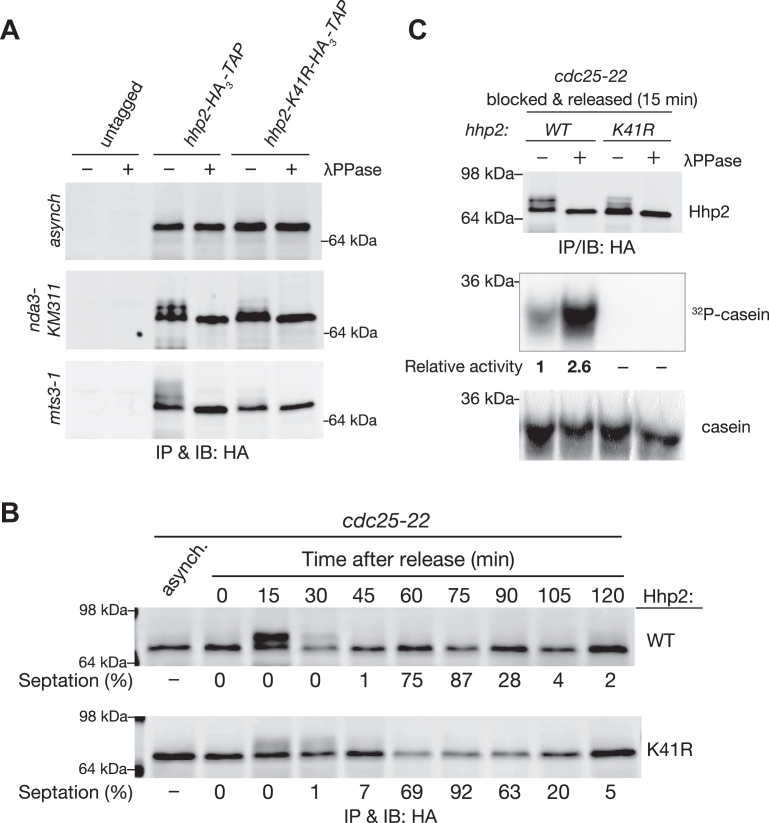
**Mitotic hyperphosphorylation of Hhp2**. *A*, protein lysates of the indicated strains that were either growing asynchronously (asynch) or arrested in mitosis (*nda3-KM311* and *mts3-1*) were subjected to anti-HA immunoprecipitation (IP). One half of each IP was treated with λ-phosphatase (λPPase) and the other with vehicle control, and proteins were immunoblotted with anti-HA (12CA5) antibodies. The position of molecular size standards is indicated to the right of the blots. *B*, *cdc25-22 hhp2-HA*_*3*_*-TAP* cells were synchronized in G2 and released into mitosis. Cell division progression was monitored by determining the percentage of septated cells over time. At times indicated, cell extracts were subject to anti-HA IP. Hhp2-HA_3_-TAP proteins were immunoblotted with anti-HA (12CA5) antibodies. The positions of molecular size standards are indicated. *C*, *cdc25*-*22* cells (15 min after releasing) from (*B*) were subjected to anti-HA IPs as in (*A*). One half of each IP was treated with λPPase and the other with buffer control, and kinase assays were performed using the bead-bound Hhp2 proteins and equivalent amounts of α-casein as substrate. Immunoprecipitated Hhp2 was visualized by immunoblot with anti-HA (12CA5) antibody and phosphorylated casein was detected by autoradiography. The relative protein kinase activities relative to λPPase-untreated Hhp2-WT are indicated. Casein was visualized by Coomassie blue stain.

We previously noted that endogenous CK1δ was apparently hyperphosphorylated in cells arrested in mitosis (33). Thus, we investigated if Hhp1 and Hhp2 are similarly phosphorylated during this cell cycle stage. Hhp1 and Hhp2, tagged at their endogenous loci with FLAG_3_ and HA_3_-TAP, respectively, were isolated from mitotically-arrested cells and treated with or without phosphatase. The strains used to arrest cells in mitosis were *nda3-KM311*, containing a mutation in β-tubulin that prevents spindle formation (55), and *mts3-1*, a mutation in a proteasome subunit that prevents anaphase (56). Only a small portion of Hhp1 was phosphorylated, and this could only be easily detected when Phos-tag (57) was included in the gels (Fig. S1). Further, Hhp1 phosphorylation was not mitosis-specific (Fig. S1, left). In contrast, we found that Hhp2-HA_3_-TAP was highly phosphorylated in both mitotically-arrested strains, as detected by the slower mobility forms on SDS-PAGE, but slower mobility forms were barely detectable in asynchronously growing cells that are primarily in interphase (Fig. 1*A*). A kinase-dead mutant, Hhp2-K41R (39), was also phosphorylated in mitotically-arrested cells, although not as extensively as wild type Hhp2, indicating that Hhp2 is both autophosphorylated and phosphorylated by another kinase(s).

To determine whether Hhp2 is also hyperphosphorylated during an unperturbed mitosis, we synchronized the cells at the G2/M boundary using the *cdc25*-*22* mutant (58), released them into mitosis, and followed Hhp2 phosphorylation status as the cells progressed through the cell cycle. Cell cycle progression was determined by monitoring the septation index, which marks the completion of mitosis and cytokinesis. Hhp2 and Hhp2-K41R became highly phosphorylated in early mitosis and dephosphorylated as cells progressed through cell division (Fig. 1*B*).

As described above, phosphorylation of CK1 enzymes often inhibits them (2, 3, 10, 12, 15, 27, 59). To determine if Hhp2 mitotic phosphorylation affects its kinase activity, we isolated hyperphosphorylated Hhp2 from cells that had been released from the G2/M block for 15 min, as in Figure 1*B*, and treated it with λ protein phosphatase or buffer alone prior to an *in vitro* kinase assay. Phosphatase treatment enhanced Hhp2 activity toward casein about 2.6-fold (Fig. 1*C*). These data indicate that during mitosis, the kinase activity of Hhp2 is attenuated *via* phosphorylation.

### Hhp2 is autophosphorylated on four inhibitory residues in the C-terminus

**Figure 2:**
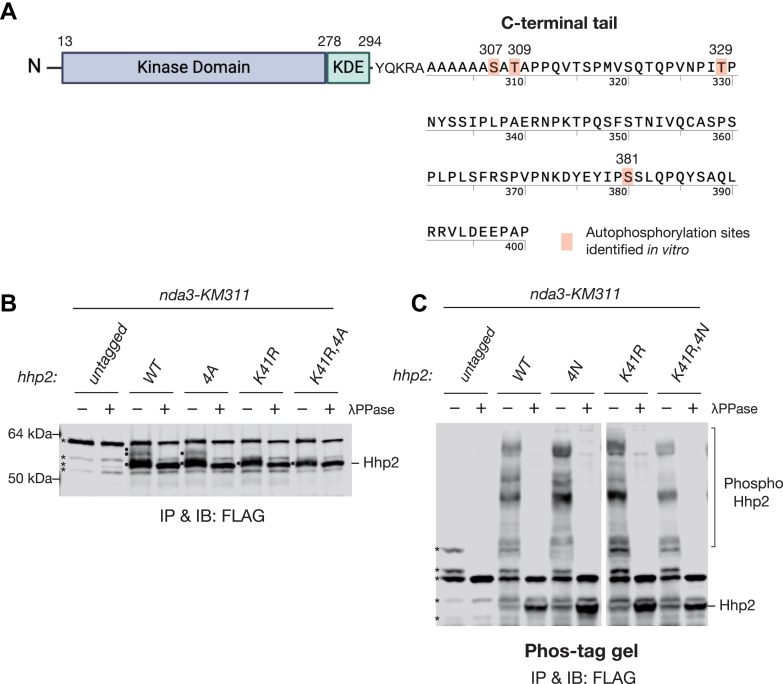
**Identification of Hhp2 autophosphorylation sites**. *A*, schematic illustration of the Hhp2 N-terminal domain structure and the C-terminal tail sequence. Major autophosphorylation sites identified *in vitro* are shown as *red-shaded* characters. KDE: kinase domain extension. *B* and *C*, *nda3-KM311* cells and *nda3-KM311* cells expressing FLAG_3_-tagged Hhp2 proteins were arrested in mitosis. Protein lysates of the indicated strains were subjected to anti-FLAG (M2) IP. One-half of each IP was treated with λPPase and the other with vehicle control, and proteins were immunoblotted with anti-FLAG M2 antibodies. 30 μM Mn^2+^–Phos-tag gel was used for separating phosphorylated species of Hhp2-FLAG more clearly in (*C*). *Asterisks* indicate non-specific bands, and solid dots and bracket indicate the positions of phosphorylated forms of Hhp2.

To investigate the consequences of Hhp2 mitotic phosphorylation *in vivo*, we sought to identify and mutate the phosphorylation sites. Since part of the Hhp2 mitotic phosphorylation we observed was due to autophosphorylation, we began by identifying these sites, which could potentially also be targeted by other kinases. We purified MBP-tagged Hhp2, and because it autophosphorylates in bacteria, treated it with λ phosphatase. Then, a portion was allowed to autophosphorylate in the presence of γ-^32^P-ATP (Fig. S2*A*). Using a classic phosphopeptide mapping approach, we found that ^32^P-labeled MBP-tagged Hhp2 gave rise to four major phosphopeptides (Fig. S2*B*, left panel). Using information annotated from global phosphoproteomic screens of *S*. *pombe* (60), and phosphopeptide mapping of individual and combination alanine site mutants, we determined that the major auto-phosphorylation sites were S307, T309, T329, and S381 (Fig. 2*A* and S2, *B* and *C*). When these residues were all mutated to alanines to generate Hhp2-4A, the major phosphopeptides were abolished (Fig. S2*B*, middle panel). As anticipated, MBP-Hhp2-4A showed enhanced kinase activity (Fig. S2*D*), to approximately the same extent as dephosphorylated endogenous Hhp2 (Fig. 1*C*).

To determine if these four sites accounted for the observed Hhp2 mitotic phosphorylation and inhibition *in vivo*, the *hhp2* gene was replaced with *hhp2-4A* or *hhp2-4N*, with asparagine rather than alanine substitutions at the four autophosphorylation sites, and tagged with FLAG_3_, an epitope that does not contain any phosphorylatable residues. The asparagine substitutions were made in case the alanine substitutions led to a loss-of-function phenotype, as was observed for *hhp1* C-terminal phosphomutants (12). Asparagine substitutions maintain the polarity of serine and threonine and loss of polarity by substituting with alanine might destabilize local hydrogen bonding and structure. Unlike for *hhp1*, we did not observe any differences in growth between the *hhp2* 4A and 4N mutants and used the strains interchangeably; they also both grew similarly to wild-type cells and were not sensitive to the DNA-damaging agent, hydroxyurea, as are *hhp2Δ* cells (Fig. S3*A*). The abundance of the Hhp2-4A mutant protein was also comparable to that of wildtype Hhp2 (Fig. 2*B*). Unexpectedly, Hhp2-4A and Hhp2-4N were still highly phosphorylated, similar to Hhp2-WT (Fig. 2, *B* and *C*). However, Hhp2-WT reproducibly generated slightly stronger slower migrating bands on SDS-PAGE and migrated with more smeared bands on Phos-tag SDS-PAGE, which were not identical to those of the Hhp2 mutants (Fig. 2, *B* and *C*). Even when the 4N mutations were combined with the catalytically inactive mutation, Hhp2-K41R-4N showed pronounced, upshifted bands on Phos-tag SDS-PAGE (Fig. 2*C*). These data indicate that while autophosphorylation is partially responsible for Hhp2 mitotic phosphorylation, Hhp2 is phosphorylated by another kinase(s) during mitosis.

### Hhp2 is phosphorylated by Cdk1 *in vitro* and *in vivo*

**Figure 3:**
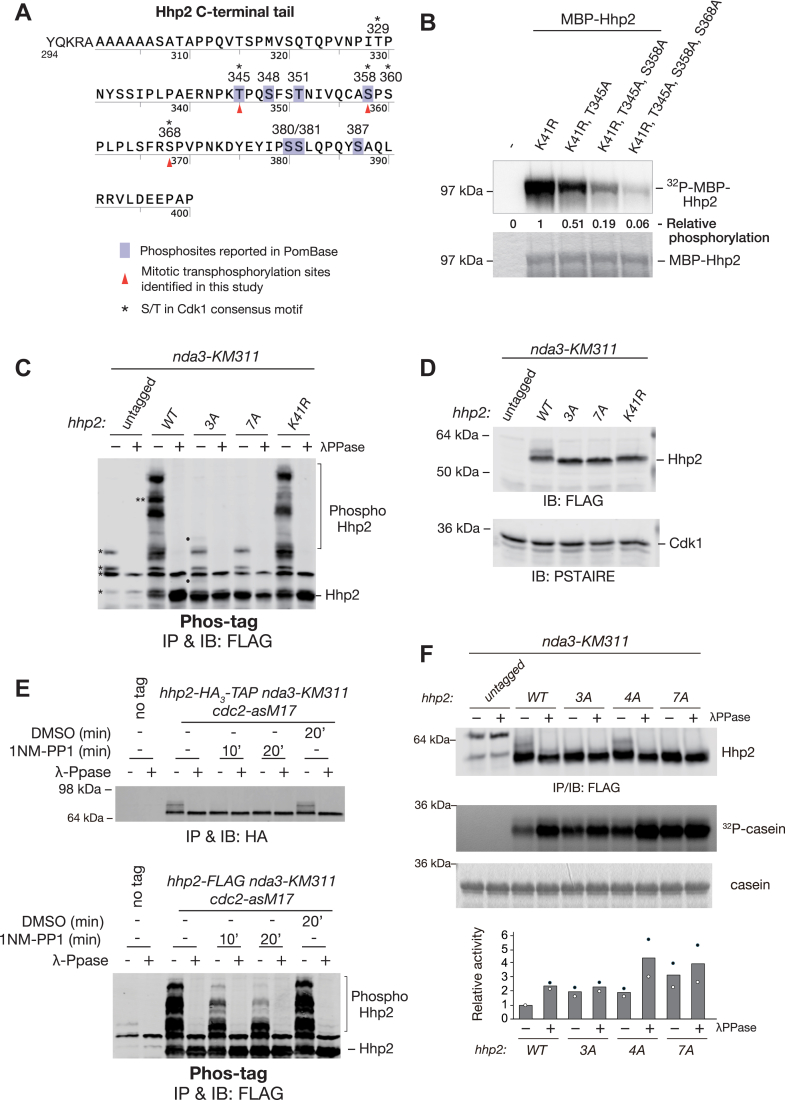
**Identification of Cdk1 phosphorylation sites in Hhp2**. *A*, potential mitotic phosphorylation sites in the C-terminal tail sequence of Hhp2 are indicated. *Purple-shaded boxes* denote the sites that had been reported as *in vivo* phosphorylation sites by global phosphoproteomic studies of *S*. *pombe*. Red triangles denote the mitotic phosphorylation sites identified in this study. *Asterisks* denote sites that fit the minimum consensus motif for Cdk1 (S/TP). *B*, *in vitro* Cdk1 kinase assays were performed using equal amounts of the indicated recombinant MBP-Hhp2 proteins as substrates. Reactions were resolved using SDS-PAGE and phosphorylated proteins were detected by phosphoimaging. The relative phosphorylation levels of Hhp2 proteins is provided under the lanes. MBP-Hhp2 was visualized by Coomassie blue stain. *C*, *nda3-KM311* cells and *nda3-KM311* cells expressing Hhp2-FLAG proteins were arrested in mitosis. Protein lysates of the indicated strains were subjected to anti-FLAG IP. One half of each IP was treated with λPPase and the other with vehicle control. Phos-tag gel was used for the separation of phosphorylated forms of Hhp2-FLAG and proteins were immunoblotted with anti-FLAG M2 antibodies. Single asterisks indicate non-specific bands and the brackets indicate phosphorylated forms of Hhp2. Solid dots in (*C*) indicate the phosphorylated bands of Hhp2-3A that disappeared in the 7A mutant. The double asterisks indicate kinase-activity-dependent phosphoshifted bands. (*D*) Protein lysates of the indicated strains were subject to immunoblotting with anti-FLAG for Hhp2 (*upper panel*) and anti-PSTAIRE for Cdk1 as a loading control (*lower panel*). *E*, *nda3-KM311 hhp2-HA*_*3*_*-TAP cdc2-asM17* (*upper panel*) and *nda3-KM311 hhp2-FLAG cdc2-asM17* (*lower panel*) cells were arrested in mitosis. They were then treated for 0, 10, or 20 min with 10 μM 1NM-PP1 or 20 min with DMSO. Protein lysates were subjected to anti-HA (*upper panel*) or anti-FLAG IP (*lower panel*). One-half of each IP was treated with λ-PPase and the other with vehicle control. Immunoprecipitated Hhp2 was visualized by immunoblot with anti-HA (*upper panel*) or anti-FLAG M2 (*lower panel*) antibody. Phos-tag gel was used for the separation of phosphorylated forms of Hhp2-FLAG. (*F*) *nda3-KM311* cells were subjected to anti-FLAG IPs. One half of each IP was treated with λPPase and the other with vehicle control, and kinase assays were performed using the bead-bound Hhp2 proteins and equivalent amounts of α-casein as substrate. Immunoprecipitated Hhp2 was visualized by immunoblot with anti-FLAG M2 antibody and phosphorylated casein was detected by autoradiography. Casein was visualized by Coomassie blue stain. The relative protein kinase activities against the λ-PPase-untreated Hhp2-WT from two independent experiments are indicated in a bar graph. *Black* and *white dots* represent each experimental data set.

As mentioned above, several *in vivo* Hhp2 phosphorylation sites have been reported from global phosphoproteomics screens of *S*. *pombe* (60) (Fig. 3*A*). We replaced these candidate sites with alanines individually and in combination in the context of the K41R catalytically inactive mutation at the endogenous Hhp2 locus, tagged the alleles with FLAG_3_, and tested for mitotic phosphorylation (Fig. S3*B*). We found that in the individual T345A and S358A mutants, the lowest mobility bands were significantly decreased, and the double mutant was even less phosphorylated (Fig. S3*C*), indicating that T345 and S358 are mitotic Hhp2 phosphorylation sites targeted by another kinase(s). However, slower mobility bands of Hhp2 were still present, evidence of remaining Hhp2 phosphorylation site(s) (Fig. S3*C*).

T345 and S358 both fit the minimum consensus sequence for Cdk1 phosphorylation (S/T-P) (61), and Cdk1 would be active at the time we observe maximal Hhp2 phosphorylation (Fig. 1*B*). We therefore asked if recombinant MBP-Hhp2-K41R could be phosphorylated by Cdk1 (in this case the active Cdc2-Cdc13 complex) (62) *in vitro*. Indeed, MBP-Hhp2-K41R was a good Cdk1 substrate (Fig. 3*B*), and MPB-Hhp2-K41R containing the T345A and S358A substitutions was still phosphorylated but to a considerably lesser extent (Fig. 3*B*). Three other Hhp2 residues lie within a Cdk1 consensus sequence: T329, S360, and S368. When S368A was combined with T345A and S358A to make Hhp2-K41R-3A, almost all Cdk1 phosphorylation was eliminated (Fig. 3*B*), indicating that Hhp2 is phosphorylated at T345, S358, and S368 by Cdk1 *in vitro*.

We next examined the phosphorylation status of Hhp2-3A and a Hhp2-7A mutant that combined the three Cdk1 sites with the four autophosphorylation sites in *nda3-KM311-*arrested cells. We replaced the wild-type *hhp2* gene with the mutants, tagged them with FLAG_3_, and detected phosphorylation-dependent mobility shifts by Phos-tag SDS-PAGE. Almost all slower mobility bands were abolished in both the 3A and 7A mutants, with a few up-shifted bands remaining in the 3A mutant (denoted with dots) that were absent in the 7A mutant (Fig. 3*C*). Thus, we had identified all significant Hhp2 mitotic phosphorylation sites. Of note, the *hhp2-3A* and *hhp2-7A* strains grew comparably to wildtype at a range of temperatures (Fig. S3*A*), and there was no effect on protein levels or cellular localization of any of these endogenous phosphosite mutations when they were tagged with either FLAG_3_ or mNeonGreen at their C-terminus (Fig.3*D* and S3*D*).

To test whether Cdk1 is responsible for Hhp2 mitotic phosphorylation in cells, we used a *cdk1/cdc2* mutant sensitive to the ATP analog 1NM-PP1, *cdc2-asM17* (63). *cdc2-asM17 nda3-KM311 hhp2-HA-TAP* and *cdc2-asM17 nda3-KM311 hhp2-FLAG*_*3*_ were arrested in mitosis and then treated with 10 μM 1NM-PP1 for 0, 10, and 20 min or vehicle control for 20 min. Inhibition of Cdk1/Cdc2 activity significantly reduced Hhp2 phosphorylation (Fig. 3*E*), confirming that Cdk1 is responsible for the majority of Hhp2 mitotic phosphorylation.

In order to see the effect of mitotic phosphorylation on Hhp2 activity, we immunoprecipitated Hhp2-FLAG_3_ mutants from mitotically arrested *nda3-KM311* cells and used the immunoprecipitates in *in vitro* kinase assays (Fig. 3*F*). Hhp2-3A and Hhp2-4A each showed increased kinase activity (∼1.9–2.0 fold). Hhp2-7A showed even higher kinase activity than either single mutant (∼3.2 fold) or dephosphorylated but autophosphorylatable Hhp2-WT (∼2.4 fold). Altogether, these results demonstrate that Cdk1-dependent phosphorylation and autophosphorylation dually inhibit Hhp2 kinase activity in mitosis.

### Hhp2 phosphorylation antagonizes its function in the Dma1-dependent mitotic checkpoint

**Figure 4:**
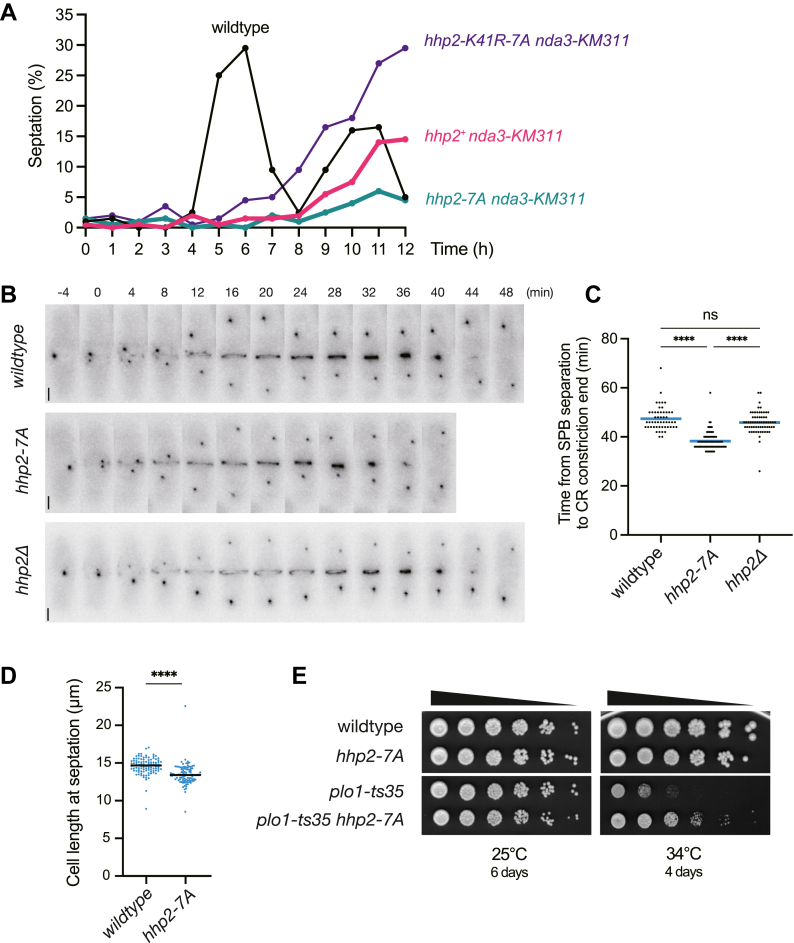
**Effects of Hhp2 phosphorylation on its mitotic functions**. *A*, the indicated strains were synchronized in S phase with hydroxyurea and shifted to 18 °C to activate the spindle checkpoint, and septation indices were measured periodically for 12 h. *B*, representative montages of live-cell time-lapse imaging of the indicated strains. SPBs (Sid4-mNG) and CR (Rlc1-mNG) are visualized. Cells were imaged every 2 min and images from every 4 min are shown. Time 0 was defined as the time of SPB separation and numbers indicate minutes from SPB separation. Scale bars: 2 μm. *C*, quantification of the duration of mitosis and cytokinesis from movies as in B. Bars represent means. n ≥ 40 cells per strain. ns, not significant (*p* = 0.9197); ∗∗∗∗, *p* < 0.0001 (one-way ANOVA with Tukey’s *post hoc* test). *D*, length at septation was quantified for indicated cells. n ≥ 100 cells. Bars represent means. ∗∗∗∗, *p* < 0.0001 by Welch’s *t* test. (*E*) Serial 5-fold dilutions of the indicated strains were spotted on YE and incubated at 25 °C or 34 °C for indicated days.

We next sought to determine if preventing Hhp2 phosphorylation affects its function in the Dma1-dependent mitotic checkpoint. To assay for checkpoint function, cells are first synchronized in S phase using hydroxyurea (HU). Following HU treatment, cells are released to the non-permissive temperature for *nda3-KM311*, and septation is monitored over time. The *hhp2* phosphomutants were not sensitive to HU in a growth assay (Fig. S3*A*), and they displayed normal mitotic onset after HU release (Fig. S4*A*), so differences in septation should solely reflect checkpoint function. While wildtype cells completed cytokinesis and septated over the time course, peaking at 5 to 6 h, *nda3-KM311* cells showed their characteristic mitotic delay, demonstrated by the longer time for septated cells to appear (9–12 h) (Fig. 4*A*). Consistent with Hhp2’s required function in the Dma1-dependent checkpoint (36, 39), catalytically inactive *nda3-KM311 hhp2-K41R-7A* cells were not able to hold the checkpoint arrest as long as *nda3-KM311* cells (Fig. 4*A*). In contrast, *nda3-KM311 hhp2-7A* cells held the arrest longer than *nda3-KM311* cells (Fig. 4*A*). This result is consistent with our *in vitro* data showing that Hhp2-7A has increased catalytic activity compared to wildtype Hhp2 and demonstrates that mitotic phosphorylation of Hhp2 inhibits its function in the mitotic checkpoint.

### Phosphorylation of Hhp2 decelerates the kinetics of unperturbed mitosis and cytokinesis

To investigate whether preventing Hhp2 phosphorylation affects progression through mitosis in the absence of a checkpoint arrest, we performed live-cell time-lapse imaging. Specifically, we compared the kinetics of mitosis and cytokinesis of *wildtype*, *hhp2-7A* or *hhp2Δ* cells by monitoring the localization of Sid4-mNG and Rlc1-mNG as markers of the SPB and the cytokinetic ring (CR), respectively. Unexpectedly, the duration of mitosis and cytokinesis (from SPB separation that indicates the onset of mitotic spindle formation to the completion of CR constriction) was accelerated in *hhp2-7A* compared to the wildtype (∼9 min, ∼1.24-fold faster) (Fig. 4, *B* and *C*), although *hhp2Δ* showed similar kinetics to wildtype. In the *hhp2-7A* mutant, each step in cytokinesis (formation of the CR, CR maturation—the time between the completion of CR formation and when constriction begins, and CR constriction) was faster (Fig. S4*B*). We also found that the cell length of septated *hhp2-7A* cells was ∼1 μm shorter than that of wildtype cells (Fig. 4*D*), suggesting a shortened G2 phase in *hhp2-7A* cells. These observations indicate that Hhp2 promotes cell proliferation, particularly when it is relieved of mitotic inhibition.

Hhp1 and Hhp2 have previously been implicated in mitotic regulation. About 30% of *hhp1Δ hhp2Δ* cells have monopolar spindles and underdeveloped spindle microtubules (38). Also, while *plo1Δ* cells are inviable in rich media, they can survive when grown in low glucose conditions, but their viability depends on the presence of Hhp1 (38). These findings suggested that Hhp1/2 ordinarily play a role in microtubule regulation during mitosis, possibly in parallel with Plo1, and raise the possibility that such a masked mitotic function of Hhp2 emerges as a phenotype in the *hhp2-7A* gain-of-function mutant. To test this hypothesis, we investigated a genetic interaction between *hhp2* and *plo1*. We observed a negative genetic interaction between *hhp2Δ* and the temperature-sensitive *plo1-ts35* allele at 32 °C, the semi-restrictive temperature (Fig. S4*C*). Furthermore, *hhp2-7A* suppressed the temperature sensitivity of *plo1-ts35* at 34 °C (Fig. 4*E*). These genetic data support the idea that Hhp2 plays a role in promoting mitosis, likely in parallel with Plo1, and such a Hhp2 function was unmasked by preventing inhibitory phosphorylation.

## Discussion

**Figure 5:**
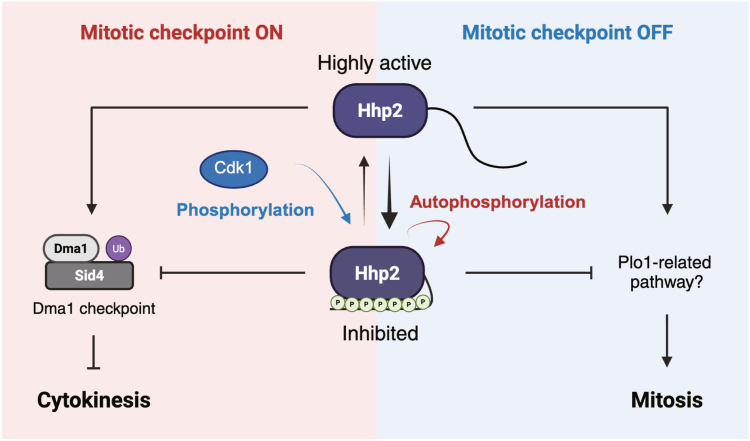
**A model of the phosphoregulated functions of Hhp2**. Hhp2 acts in the mitotic checkpoint activity by phosphorylating the Sid4 scaffold. That function is attenuated by its hyperphosphorylation, mediated by a combination of Cdk1 phosphorylation and autophosphorylation. Outside of an imposed mitotic arrest, Hhp2 functions to accelerate cell division. This function, which suppresses the need for Plo1 kinase activity, is also attenuated by mitotic phosphorylation. Created with BioRender (www.biorender.com).

CK1 C-terminal phosphorylation and the resultant intramolecular inhibition are distinguishing features of these enzymes; however, hyperphosphorylation of these proteins has rarely been observed in cells or tissues. Here, we showed that one of the CK1δ/ε-related CK1 enzymes in *S*. *pombe* is transiently hyperphosphorylated in a physiologically relevant situation, during mitosis, by a combination of Cdk1-mediated phosphorylation and autophosphorylation. We further found that mitotic phosphorylation of Hhp2 inhibited its kinase activity *in vitro* and its mitotic checkpoint function *in vivo*. By constitutively removing this negative regulation *via* mutation, we also unmasked a role of Hhp2 in driving cell proliferation, likely in parallel with the Plo1 kinase (Fig. 5).

### Mechanism by which mitotic phosphorylation sites affect Hhp2 kinase activity

In this study, we identified four autophosphorylation sites and three Cdk1 sites within Hhp2. However, it remains to be determined whether or not Hhp2 is phosphorylated at all these sites simultaneously or if there are any phosphorylation patterns that are sufficient to inhibit its *in vivo* function. We also do not know if any one phosphorylation event affects the phosphorylation of another, potentially resulting in a phosphorylation cascade. This is an interesting possibility given that CK1 enzymes often target primed substrates (64, 65), and we indeed observed kinase-activity-dependent phosphoshifts by Phos-tag SDS-PAGE that disappeared in the Hhp2-3A mutant (Fig. 3*C*). Regardless, our data indicate that not only Cdk1-dependent phosphorylation but also Hhp2 autophosphorylation contributes to inhibiting Hhp2 function in the mitotic checkpoint and that preventing these phosphorylation events leads to a gain-of-function mutant. Hhp2 auto-inhibition by phosphorylation at multiple sites throughout the C-terminus might be analogous to the situation for CK1δ, in which any one of a number of C-terminal phosphorylation sites can bind and inhibit the CK1δ catalytic domain (10). Alternatively, the relative localization of phosphorylation sites might be critical to inhibit Hhp2 function, as shown for the effect of autophosphorylation of the unique C-terminus of the CK1δ1 isoform (15). AlphaFold3 (66) generated a set of five models with similar confidence scores [(∼0.7 predicted template modelling score (pTM)], in which the phosphorylated C-terminus interacts with the catalytic domain of Hhp2 (Fig. S5, *A* and *C* show two of the models). In the different models, different phosphorylation sites (pT329, pT345, pS358, and pS381) were predicted to interact with G216, R179, and K225, which form Site 1, while pS368 or pS358 were predicted to reside in the Site 2 pocket formed by residues R130, K155, and K172 (Fig. S5, *B* and *D*). While these models are consistent with the idea that multiple C-terminal phosphorylation sites are capable of inhibiting Hhp2, future studies will be required to confirm the significance of these structural predictions and to elucidate how the phosphorylated C-terminus docks within the substrate binding groove.

### Physiological significance of Hhp2 phosphoregulation in mitosis

The present findings show that the mitotic function of Hhp2 in the Dma1-dependent checkpoint is inhibited through C-terminal domain phosphorylation (Fig. 5). Thus, Hhp2 hyperphosphorylation seems likely to be a checkpoint silencing mechanism. In this context, it is interesting to note that while both Hhp1 and Hhp2 are required for delaying cytokinesis when the mitotic spindle is disrupted, Hhp2 appears to play the more dominant role (36), and it may be especially important to attenuate its activity to allow recovery from the arrest. This would require fine-tuned temporal regulation of Hhp2 phospho-state, in which Hhp2 would be dephosphorylated when the checkpoint is on, then phosphorylated once the checkpoint is satisfied to allow cell cycle progression. Therefore, understanding the action of the phosphatase(s) that respond to the checkpoint signal would enhance our understanding of checkpoint initiation and silencing. Phosphatases that are active during mitosis, such as PP1 and PP2A, have been shown to dephosphorylate mammalian CK1δ/ε (9, 27, 32) and perhaps the same occurs in *S*. *pombe*.

Given that *hhp2-7A* behaved as a gain-of-function mutant in the Dma1-dependent checkpoint, holding the arrest longer than wildtype, we were surprised that *hhp2-7A* cells were shorter and accomplished cell division faster than wildtype in the absence of prolonged checkpoint stimulation. This suggests that the substrate(s) targeted by Hhp2 during an unperturbed mitosis may be different than those targeted during the checkpoint (Sid4). C-terminal phosphorylation of CK1 enzymes does not completely inhibit them but rather can shift substrate selectivity (10, 12). Consequently, it is possible that while the target of Hhp2 in the checkpoint is phosphorylated better by Hhp2-7A, other substrates might fail to be phosphorylated at the same time. Thus, it will be interesting to determine how the substrate profile of Hhp2-7A compares to that of wildtype, as well as to identify the relevant substrates through which Hhp2 promotes mitosis and cytokinesis.

### Conservation of mitotic phosphoregulation among CK1 enzymes

Mitotic hyperphosphorylation of endogenous and overproduced human CK1δ has been observed in HeLa and U2OS cells (33, 34), and studies using Cdk inhibitors have also suggested that CK1δ is inhibited by Cdk activity (24, 25). One report has suggested that mitotic phosphorylation might lead to degradation of excess CK1δ (34). However, we did not detect any effects of phosphorylation or lack thereof on the protein stability of Hhp2, and we therefore favor a model whereby Cdk1-mediated phosphorylation leads to catalytic inhibition (Fig. 5) rather than protein destruction. In any case, Cdk1-mediated phosphorylation and inhibition of CK1 activity is likely to be a conserved mechanism of coupling the core cell cycle machinery to CK1 functions. As we observed mitotic phosphoregulation for Hhp2 but not for Hhp1, it will be interesting to determine if CK1δ/ε and CK1α are also differentially phosphorylated during the cell cycle to modulate their kinase activity. This mechanism could provide divergent regulatory inputs to enzymes that otherwise share overlapping functions.

## Conclusion

In this study, we revealed that a CK1 enzyme in fission yeast, Hhp2, is hyperphosphorylated during mitosis and that the master cell cycle kinase Cdk1 participates in this phosphoregulation, firmly connecting two kinase families together in a signaling network. Together with a previous report that the paralog of Hhp2, Hhp1, can replace the function of the Plo1 kinase, our data highlight the need for additional investigation of the mechanisms by which CK1 enzymes participate in cell cycle control. Furthermore, because we discovered that phosphoregulation of a CK1 enzyme has a biological impact during mitosis, this suggests that phosphoregulation of other CK1 family members in other cellular contexts may also be meaningful. Therefore, understanding this regulatory mechanism may have a broader impact in areas such as disease-related CK1 signaling.

## Experimental procedures

### Yeast methods

*S*. *pombe* strains used in this study (Table S1) were grown in yeast extract with supplements (YE) at 25 °C or 32 °C unless otherwise indicated. Genetic crosses were performed and analyzed as described (67). *hhp1* and *hhp2* were tagged endogenously at the 3′ end of their open reading frames (ORFs) with *HA*_*3*_*-TAP*:*kanMX6*, *FLAG*_*3*_:*kanMX6*, or *mNeonGreen*:*kanMX6* using pFA6 cassettes as previously described (68). G418 (100 μg/ml) (Sigma-Aldrich) in YE media was used for selecting *kanMX6* cells. A lithium acetate transformation method (69) was used for introducing tags, which were confirmed by whole-cell PCR and Western blotting or microscopy. *hhp2* point mutants were generated in pCR-blunt constructs using site-directed mutagenesis, and amplified *hhp2* fragments with 500 bp flanks were subsequently integrated over the null (*hhp2Δ*:*:ura4*^*+*^) using a lithium-acetate protocol. Integrants were selected on YE plates containing 1.5 mg/ml 5-fluoroorotic acid (FOA; Toronto Research Chemicals) and validated by colony PCR, followed by DNA sequencing.

For *nda3-KM311* arrests, log-phase (OD_595_ 0.2–0.8) cells cultured at 32 °C were shifted to 18 °C for 5 or 6 h to arrest them in mitosis. *nda3-KM311 cdc2-asM17* cells were then treated with 10 μM 1-(1,1-dimethylethyl)-3-(1-naphthalenylmethyl)-1H-pyrazolo[3,4-days]pyrimidin-4-amine (1-NM-PP1; Cayman Chemical) for 0, 10 and 20 min or DMSO for 20 min before sample collection. For *mts3-1* arrests, log-phase cells cultured at 25 °C were shifted to 36 °C for 3 h. For *cdc25*-*22* block-and-release experiments, log-phase cells cultured at 25 °C were arrested in G2 phase at 36 °C for 3.5 h and then released to 25 °C at the 0 time point before sample collection.

For growth assays, three 10-fold serial dilutions or five 5-fold serial dilutions were made in sterile water starting at 4.0 x 10^6^ cells/ml. 3 μl of each dilution was spotted on YE plates and cells were grown at the indicated temperatures for the indicated days.

### Checkpoint assay

*S*. *pombe* cells were synchronized in S phase using hydroxyurea (HU) (Sigma) at a final concentration of 12 mM for 3 to 3.5 h at 32 °C. Cells were then filtered into HU-free and pre-cooled YE media and immediately incubated at 18 °C to activate the spindle checkpoint. Septation indices were measured periodically for 12 h by live observation with a microscope. For the HU release assay, cells were fixed every 30 min and subsequently a SPB marker was imaged to determine the kinetics of mitotic onset and progression (described in Microscopy methods). Septation indices were also determined in live cells and monitored every 15 min using a microscope.

### Immunoprecipitation (IP) and Western blotting

*S*. *pombe* strains were grown in YE to log phase. 25 OD_595_ cell pellets were washed once in NP-40 buffer [10 mM NaPO_4_ pH 7.0, 1% NP-40, 150 mM NaCl, 2 mM EDTA, 50 mM NaF, 4 μg/ml leupeptin, 100 mM Na_3_VO_4_, 1 mM PMSF, 1.3 mM benzamidine, protease inhibitor tablets (Roche)] and lysed by bead disruption using a FastPrep cell homogenizer (MP Biomedicals). The homogenized cell/bead mixture was treated with 500 μl SDS lysis buffer [10 mM sodium phosphate, pH 7.0, 1% SDS, 1 mM DTT, 1 mM EDTA, 50 mM NaF, 100 μM sodium orthovanadate, 1 mM PMSF, 4 μg/ml leupeptin, and 1x PhosSTOP (Roche)] and incubated at 95 °C for 2 min. Lysate was extracted with 800 μl NP-40 buffer containing 1x PhosSTOP and cleared by centrifugation at 17,000 x *g* for 5 min at 4 °C, followed by further centrifugation at 17,000 x *g* for 20 min at 4 °C. Hhp2-HA_3_-TAP was immunoprecipitated using 1 μg anti-HA antibody (12CA5) (Vanderbilt Antibody and Protein Resource) at 4 °C for 1 h, followed by the addition of Protein A Sepharose beads (GE Healthcare) for 30 min Hhp1-FLAG_3_ was immunoprecipitated using 2 μg anti-FLAG antibody M2 (Sigma) at 4 °C for 1 h, followed by the addition of Protein G Sepharose beads for 30 min Hhp2-FLAG_3_ was immunoprecipitated using 12.5 μl of DYDDDDK Fab-Trap Agarose (Chromotek) at 4 °C for 1 h. For the phosphatase treatment, the beads with Hhp1 or Hhp2 were equilibrated in 1x Protein Metallo Phosphatase (PMP) buffer (New England Biolabs) and then split into two tubes. One half was incubated with 1.5 μl of λ protein phosphatase (λPPase) (New England Biolabs) in the presence of 10 mM MnCl_2_ for 45 min at 30 °C. For control, buffer only was added to the other half of the immunoprecipitate. After washing the beads with PMP buffer or NP-40 buffer, proteins were eluted by boiling in 30 μl SDS-PAGE sample buffer. Samples were separated by SDS-PAGE and/or 20 to 30 μM Mn^2+^–Phos-tag SDS-PAGE (57), transferred to Immobilon-P polyvinylidene fluoride membrane (Millipore) at 30 V for 1 h (1.5 h for Phos-tag gel), immunoblotted with mouse anti-FLAG M2 or mouse anti-HA (12CA5) and fluorescent IRDye 680LT goat anti-mouse IgG antibody (Li-Cor Biosciences), and imaged on an Odyssey CLx (Li-Cor Biosciences).

### Molecular biology and protein purification

All plasmids used in the study were generated by standard molecular biology techniques. For protein production of MBP-Hhp2 and MBP-Hhp1 from bacteria, pMAL-c2-Hhp1 and pMAL-c2-Hhp2 were used (12). Mutagenesis was performed using QuikChange site-directed mutagenesis kit (Agilent Technologies). Plasmids were validated by DNA sequencing.

MBP fusion protein production was induced in *Escherichia coli* Rosetta2(DE3)pLysS cells (Novagen), and cell lysate was prepared as previously described (12). MBP fusion proteins were purified on Hi-Trap MBP column (1 ml) (Cytiva) in column buffer (20 mM tris (pH 7.4), 150 mM NaCl, 1 mM EDTA, 0.1% NP- 40, 1 mM dithiothreitol (DTT), 1 mM PMSF, 1.3 mM benzamidine, protease inhibitor tablets) and eluted with the column buffer containing 10 mM maltose and 10% glycerol. Protein purification was done by AKTApure M25 system (Cytiva).

### *In vitro* kinase assays

For *in vitro* kinase assays on immunoprecipitated Hhp2-HA-TAP, the denaturing procedure using SDS lysis buffer was omitted and proteins were directly extracted by adding 800 μl NP-40 buffer containing 1x PhosSTOP. After immunoprecipitation, the beads were equilibrated in 1x PMP buffer (New England Biolabs). Before kinase reactions, Hhp2 was dephosphorylated by 1.5 μl of λPPase in the presence of 10 mM MnCl_2_ for 45 min at 30 °C. For control, the vehicle was added to the reaction instead of λPPase. After washing out the phosphatase with 1x PMP buffer twice and kinase reactions were performed with 15 μg of α-casein (Sigma) in 1x PMP buffer supplemented with 100 μM unlabeled ATP, 3 μCi γ-^32^P-ATP, and 10 mM MgCl_2_ at 30 °C for 30 min.

Phosphorylation of MBP-Hhp2-K41R mutants (1.32 μg) was performed in PMP buffer with 100 μM cold adenosine triphosphate (ATP), 2 μCi of γ-^32^P-ATP, active Cdc13-Cdk1 prepared from insect Sf9 cells (62), and 10 mM MgCl_2_ for 40 min. The kinase reactions were quenched by boiling in SDS-PAGE buffer, and separated by SDS-PAGE. Subsequently, the gel was stained with Coomassie Blue, dried, and phosphoproteins were detected by autoradiography. ^32^P was quantified on a Typhoon FLA 7000 phosphoimager. The relative protein kinase activity was calculated relative to the λPPase-untreated Hhp2 activity for each experiment. The kinase activities for casein were normalized for each mutant.

### Phosphopeptide mapping

Prior to autophosphorylation reaction, recombinant MBP-Hhp2 (4 μg) was dephosphorylated by 2 μl of λPPase in the presence of 10 mM MnCl_2_ for 30 min at 30 °C. Phosphatase reactions were quenched by the addition of 1x PhosSTOP (4 μl). The following autophosphorylation reactions and phosphopeptide mapping were conducted as previously described (12).

### Microscopy methods

Images of live *hhp2-mNeonGreen* cells and 70% ethanol-fixed *sid4-mNeonGreen* cells were acquired using a Zeiss Axio Observer inverted epifluorescence microscope which includes an AxioCam 503 mono camera with Zeiss Plan Apochromat 63x oil (1.46 NA) objective and captured using Zeiss ZEN 3.0 (Blue edition) software. The z-stack step size was 0.5 μm and a total of 10 Z-slices were acquired. Images for *hhp2-mNeonGreen* cells were max projections. The percentage of cells containing one or two SPB(s) was counted for each cell population using Fiji software (70). More than 180 cells for each strain were imaged and counted.

Time-lapse imaging was performed using a Leica Thunder imager system including a DMI8 inverted microscope, 63X plan apo oil objective (1.40 NA), a Leica K8 sCMOS camera, standard excitation and emission filters, and an LED light source (Leica Microsystems). Images were acquired using Leica Application Suite X (LAS X) software (Leica Microsystems). A CellASIC ONIX microfluidics perfusion system (Millipore Sigma) was used, and cells were loaded into Y04 C plates for 10 s at 8 psi. YE liquid medium flowed through the chamber at 5 psi throughout imaging. Z-series optical sections were taken at 0.5 μm spacing and images were acquired every 2 min. Two biologically independent movies containing multiple cells of each strain were acquired and the cell images were combined for comparison.

### AlphaFold3 structural prediction

The phosphorylated Hhp2 structure predictions were generated with the AlphaFold3 server (https://alphafoldserver.com) (66). The automatic seed setting was used for all predictions. Figure S5 shows the top-ranked “model_0” and the second-ranked “model_1” predictions that were further analyzed using the PyMOL molecular graphics system (version 3.0, Schrodinger, LLC).

### Statistical analysis

Statistical analysis was performed using GraphPad Prism v8.

## Data availability

The raw data that support the findings of this study are openly available in Mendeley data at DOI: 10.17632/5nhgfcxbpy.1 and 10.17632/bh4v6c4pkc.1. The authors confirm that the data supporting the findings of this study are available within the article and its supplementary materials.

## Supporting information

This article contains supporting information.

## Conflict of interest

The authors declare that they do not have any conflicts of interest with the content of this article.
