# Supplementary material for: The mitotic functions of a fission yeast CK1 enzyme are regulated by Cdk1-dependent and auto-phosphorylation
Source: J Biol Chem. 2025 Dec 5;302(1):111007. doi: 10.1016/j.jbc.2025.111007 (PMC12804105; doi:10.1016/j.jbc.2025.111007)
Supplement: Supplementary Material 1 [file mmc1.pdf]

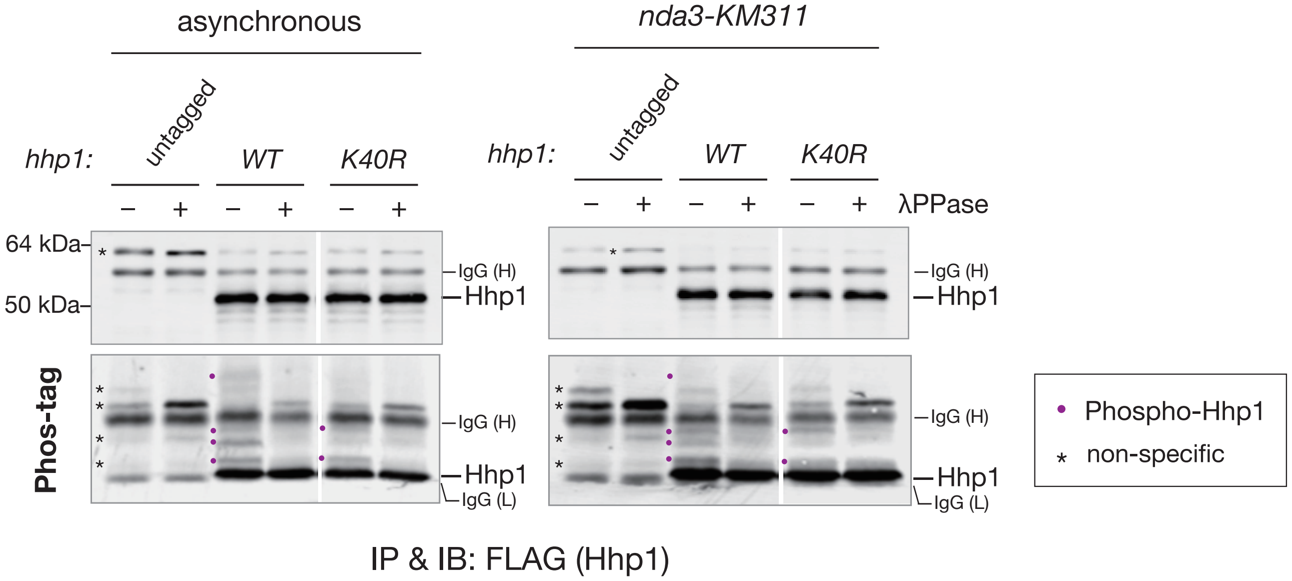
asynchronous
nda3-KM311
hhp1:
untagged
WT
K40R
hhp1:
untagged
WT
K40R
λPPase
64 kDa
50 kDa
IgG (H)
Hhp1
IgG (H)
Hhp1
Phos-tag
IgG (H)
Hhp1
IgG (L)
IgG (H)
Hhp1
IgG (L)
Phospho-Hhp1
non-specific
IP & IB: FLAG (Hhp1)

Fig.S1

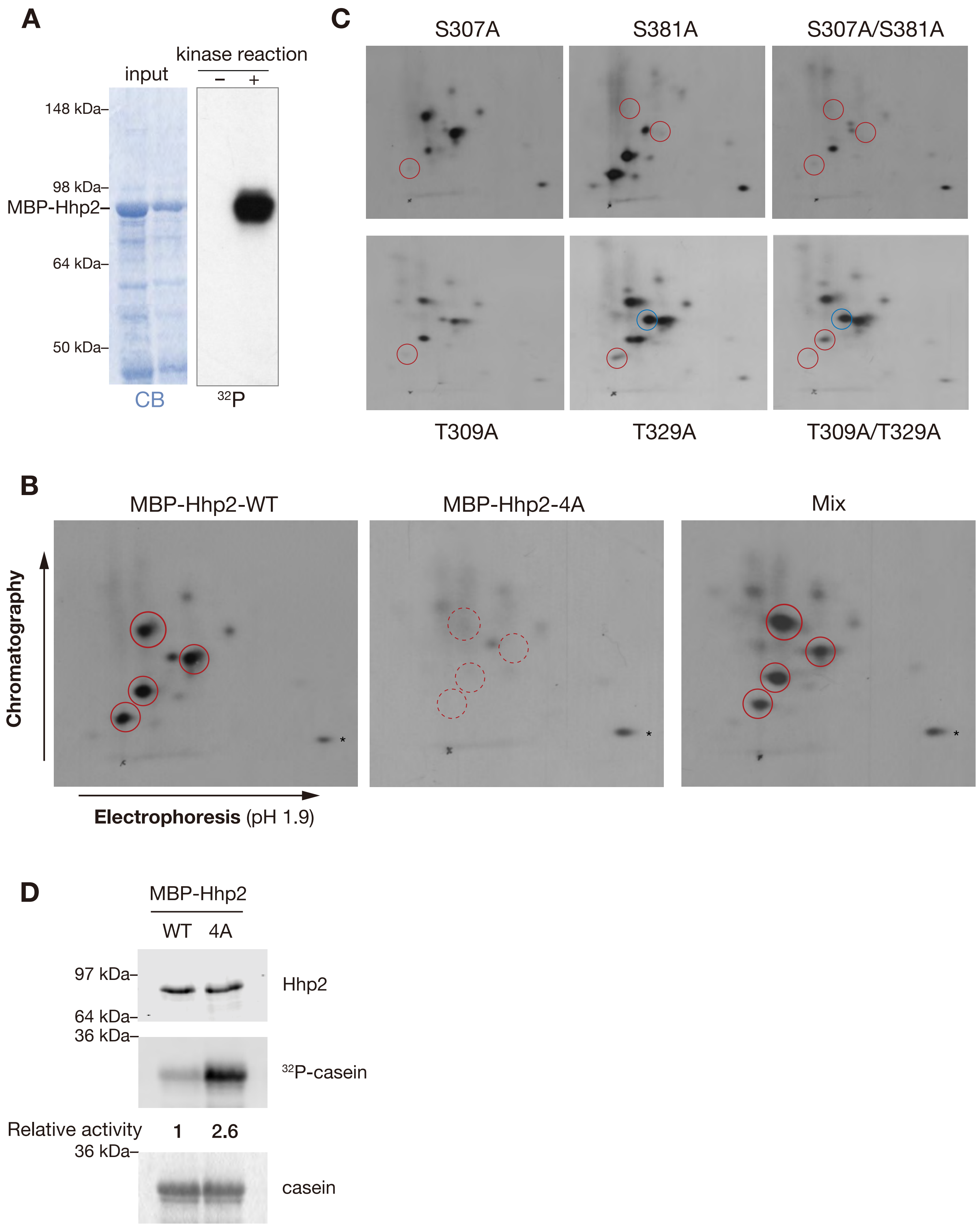
A
input
kinase reaction
– +
148 kDa–
98 kDa–
MBP-Hhp2–
64 kDa–
50 kDa–
CB
32P
C
S307A
S381A
S307A/S381A
T309A
T329A
T309A/T329A
B
MBP-Hhp2-WT
MBP-Hhp2-4A
Mix
Chromatography
Electrophoresis (pH 1.9)
D
MBP-Hhp2
WT 4A
97 kDa–
64 kDa–
Hhp2
36 kDa–
32P-casein
Relative activity 1 2.6
36 kDa–
casein

Fig. S2

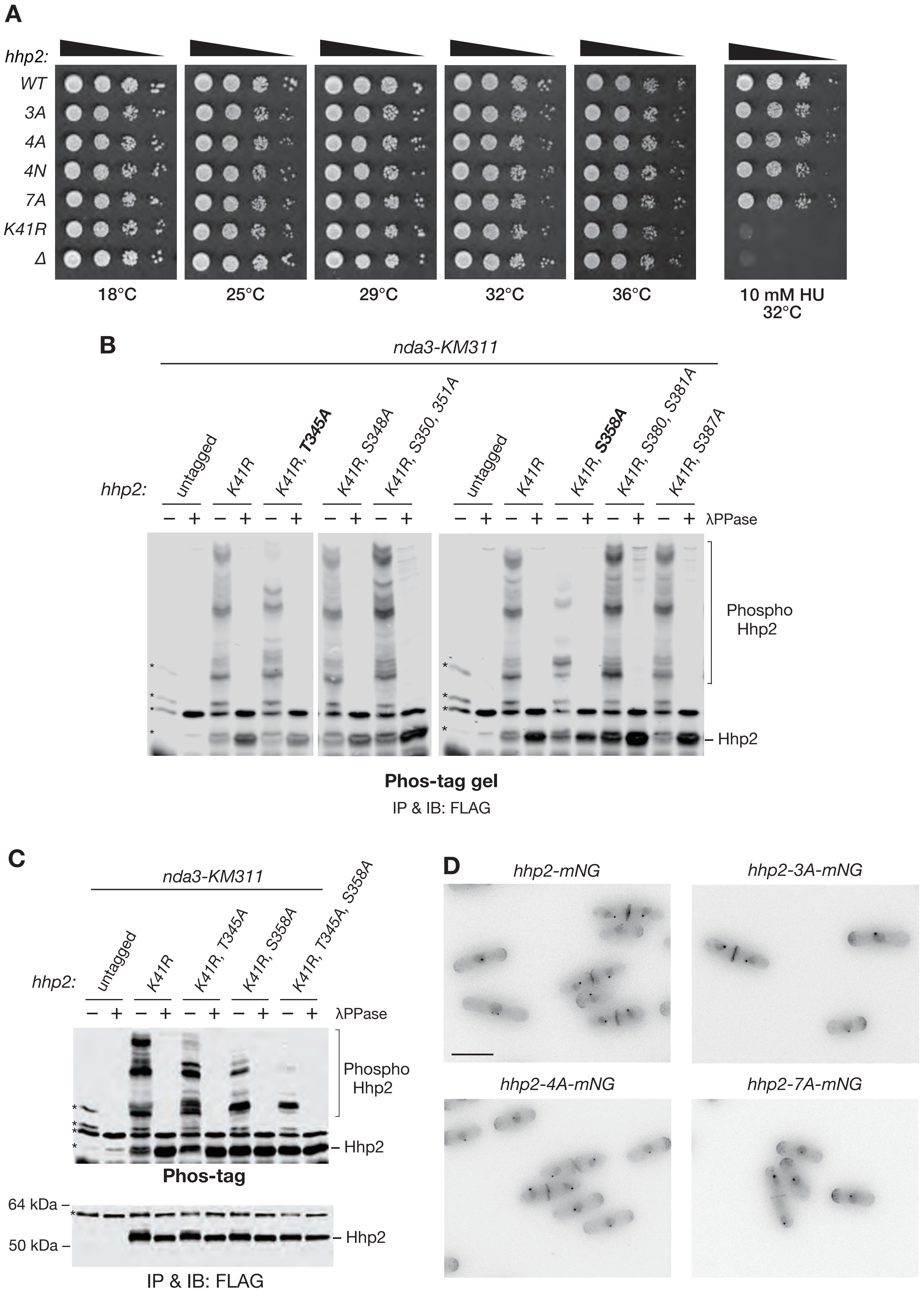
A
hhp2:
WT
3A
4A
4N
7A
K41R
Δ
18°C
25°C
29°C
32°C
36°C
10 mM HU
32°C
B
nda3-KM311
hhp2:
untagged
K41R
K41R, T345A
K41R, S348A
K41R, S350, 351A
untagged
K41R
K41R, S358A
K41R, S380, S381A
K41R, S387A
λPPase
Phospho
Hhp2
Hhp2
Phos-tag gel
IP & IB: FLAG
C
nda3-KM311
hhp2:
untagged
K41R
K41R, T345A
K41R, S358A
K41R, T345A, S358A
λPPase
Phospho
Hhp2
Hhp2
Phos-tag
64 kDa
50 kDa
Hhp2
IP & IB: FLAG
D
hhp2-mNG
hhp2-3A-mNG
hhp2-4A-mNG
hhp2-7A-mNG

Fig. S3

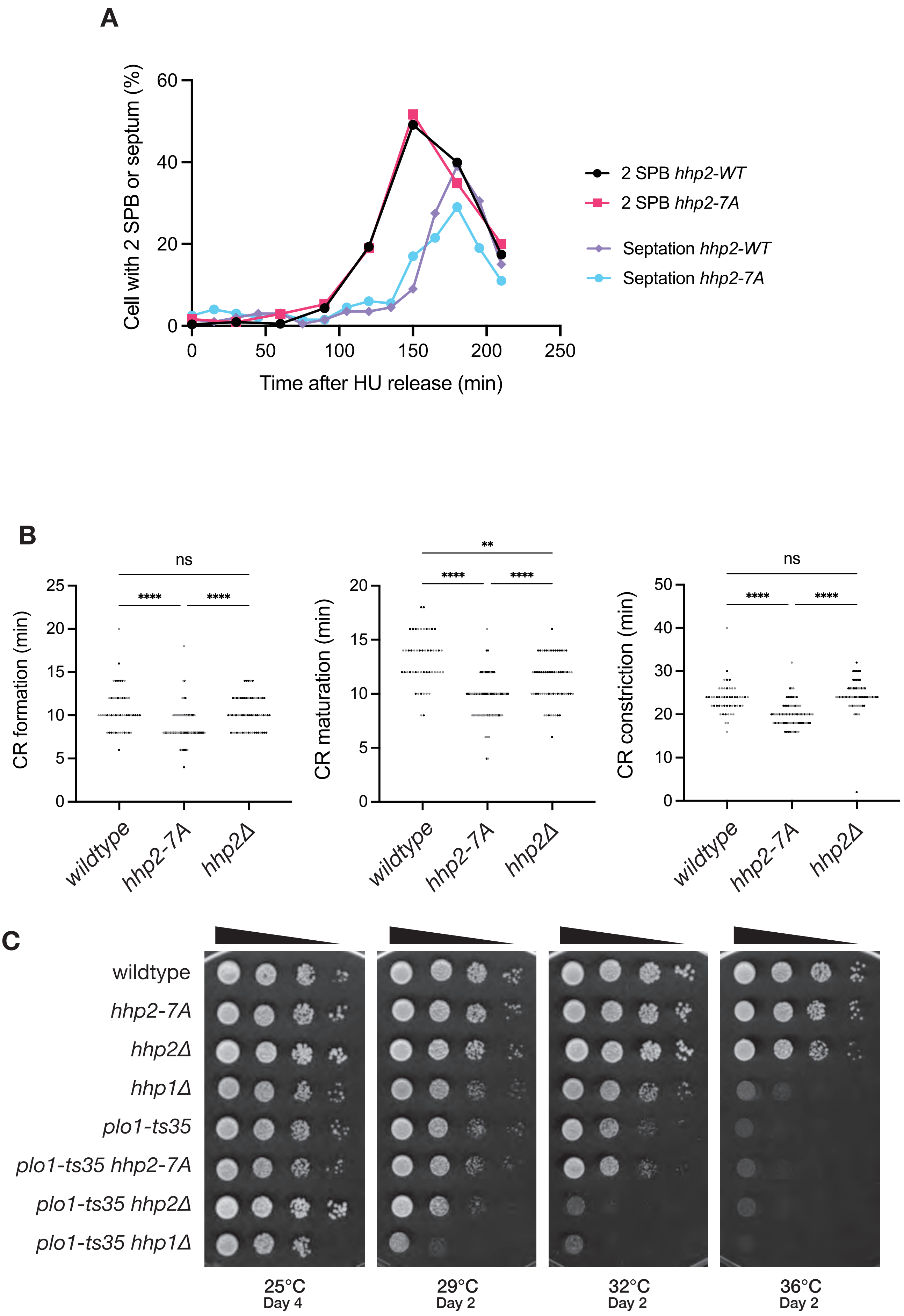
A
Cell with 2 SPB or septum (%)
60
40
20
0
0
50
100
150
200
250
Time after HU release (min)
2 SPB hhp2-WT
2 SPB hhp2-7A
Septation hhp2-WT
Septation hhp2-7A
B
ns
****
****
CR formation (min)
25
20
15
10
5
0
wildtype
hhp2-7A
hhp2Δ
**
****
****
CR maturation (min)
20
15
10
5
0
wildtype
hhp2-7A
hhp2Δ
ns
****
****
CR constriction (min)
50
40
30
20
10
0
wildtype
hhp2-7A
hhp2Δ
C
wildtype
hhp2-7A
hhp2Δ
hhp1Δ
plo1-ts35
plo1-ts35 hhp2-7A
plo1-ts35 hhp2Δ
plo1-ts35 hhp1Δ
25°C
Day 4
29°C
Day 2
32°C
Day 2
36°C
Day 2

Fig.S4

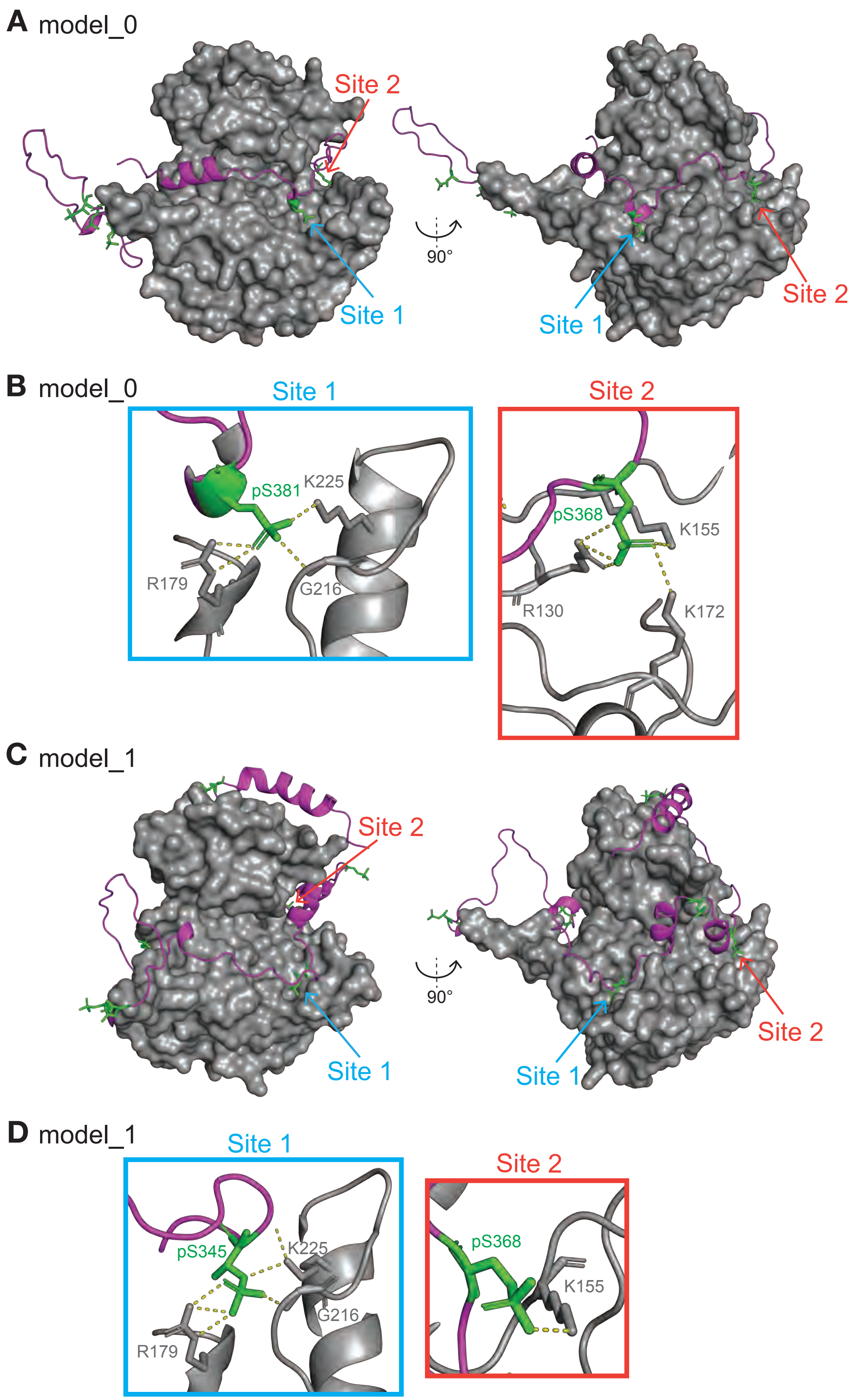

A model_0
Site 2
Site 1
90°
Site 1
Site 2
B model_0
Site 1
pS381
K225
R179
G216
Site 2
pS368
K155
R130
K172
C model_1
Site 2
Site 1
90°
Site 1
Site 2
D model_1
Site 1
pS345
K225
G216
R179
Site 2
pS368
K155

Fig.S5

Supplementary figure legends

Figure S1. Hhp1 phosphorylation does not appear to be cell-cycle regulated.

One half of anti-FLAG IPs from the indicated strains was treated with λPPase and the other with vehicle control, and proteins were immunoblotted with anti-FLAG (M2) antibody. 20 μM Mn^{2+}–Phos-tag gel was used for separating phosphorylated species of Hhp1-FLAG. The position of the heavy chain (H) of IgG is indicated.

Figure S2. Identification of Hhp2 autophosphorylation sites.

(A) Recombinant MBP-Hhp2 was dephosphorylated with λPPase (left lane) and then autophosphorylated in the presence of [γ-^{32}P]ATP. MBP-Hhp2 proteins were visualized with Coomassie and autophosphorylated MBP-Hhp2 was detected by autoradiography.

(B and C) Phosphopeptide maps of MBP-Hhp2 and its mutants. Phosphorylated peptides were detected by autoradiography. X's mark the spotting origins. (B) Red circles indicate the four major Hhp2 phosphopeptides. Blue circles: phosphopeptides that changed mobility because an amino acid was mutated within the phosphopeptide. *: a phosphopeptide arising from the MBP-tag.

(D) Recombinant MBP-Hhp2-WT and its 4A mutant were allowed to autophosphorylate at 30°C for 30 minutes. Subsequently, they were used to phosphorylate casein in the presence of [γ-^{32}P]-ATP. Phosphorylated casein was detected by autoradiography (^{32}P) and protein levels of Hhp2 (autophosphorylated) used for casein phosphorylation were visualized by Coomassie (top panel).

Figure S3. Identification of Cdk1 phosphorylation sites within Hhp2

(A) Serial 10-fold dilutions of the indicated strains were spotted on YE or on YE with 10 mM HU and incubated at indicated temperatures.
(B) Protein lysates of the indicated strains were subjected to anti-FLAG IP. One half was treated with λPPase and the other with buffer, and proteins were immunoblotted with anti-FLAG M2 antibodies. Asterisks indicate non-specific bands and the brackets indicate phosphorylated forms of Hhp2.
(C) *nda3-KM311* cells and *nda3-KM311* cells expressing Hhp2-FLAG proteins were arrested in mitosis. Protein lysates of the indicated strains were subjected to anti-FLAG IP. One half of each IP was treated with λPPase and the other with vehicle control, and proteins were immunoblotted with anti-FLAG M2 antibodies. A phos-tag gel was used for the separation of phosphorylated forms of Hhp2-FLAG (upper panel).

(D) Representative live-cell images of Hhp2-mNG and its mutants. Scale bars: 10 μm.

Figure S4.
(A) The indicated strains were synchronized in S phase with hydroxyurea at 32°C and shifted to 25°C and further cultured for 3.5 h. The percentages of cells with 2 SPBs and septated cells are indicated.
(B) Quantification of the duration of each stage of mitosis from movies in Fig. 4C. Bars represent means. $n \geq 40$ cells per strain. ns, not significant; ****, $P < 0.0001$; ***, $P<0.001$; **, $P<0.01$ (one-way ANOVA with Tukey's post-hoc test).
(C) Serial 10-fold dilutions of the indicated strains were spotted on YE and incubated at indicated temperatures.

Figure S5. the Hhp2 C-terminal tail is predicted to bind multiple sites on the kinase domain. (A and C) AlphaFold3-generated top-ranked "model_0" and the second-ranked "model_1" of full-length Hhp2 with the following residues phosphorylated: S307, T309, T329, T345, S358, S368, and S381. The surface of the kinase domain is shown in gray, the C-terminal tail is represented as a ribbon in magenta, and the phosphorylated residues are in green. (B and D) Zoomed-in views of site #1 and site #2 of the kinase domain in which two phosphorylated residues are bound.

Supplementary Table 1: ***S. pombe*** **strains used in this study.**

KGY Strain #	Genotype	Source
**Figure 1**		
19334	*hhp2-K41R ade6-M210 ura4-D18 leu1-32 h*$^{+}$	This study
12947	*hhp2-HA*$_3$*-TAP:kanMX6 ade6-M210 ura4-D18 leu1-32 h*$^{-}$	This study
7323-2	*hhp2-K41R-HA*$_3$*-TAP:kanMX6 ade6-M210 ura4-D18 leu1-32 h*$^{+}$	This study
14398	*nda3-KM311 hhp2-HA*$_3$*-TAP:kanMX6 ade6-M210 ura4-D18 leu1-32 h*$^{+}$	This study
7324-2	*nda3-KM311 hhp2-K41R-HA*$_3$*-TAP:kanMX6 ade6-M210 ura4-D18 leu1-32 h*$^{+}$	This study
14401	*mts3-1 hhp2-HA*$_3$*-TAP:kanMX6 ade6-M210 ura4-D18 leu1-32 h*$^{+}$	This study
7364-2	*mts3-1 hhp2-K41R-HA*$_3$*-TAP:kanMX6 ade6-M210 ura4-D18 leu1-32 h*$^{+}$	This study
7734-2	*cdc25-22 hhp2-HA*$_3$*-TAP:kanMX6 ade6-M210 ura4-D18 leu1-32 h*$^{+}$	This study
7735-2	*cdc25-22 hhp2-K41R-HA*$_3$*-TAP:kanMX6 ade6-M210 ura4-D18 leu1-32 h*$^{+}$	This study
**Figure 2**		
7324-2	*nda3-KM311 hhp2-K41R-HA*$_3$*-TAP:kanMX6 ade6-M210 ura4-D18 leu1-32 h*$^{+}$	This study
9119-2	*nda3-KM311 hhp2-FLAG*$_3$*:kanMX6 ade6-M210 ura4-D18 leu1-32 h*$^{+}$	This study
9122-2	*nda3-KM311 hhp2-4A-FLAG*$_3$*:kanMX6 ade6-M210 ura4-D18 leu1-32 h*$^{-}$	This study
9120-2	*nda3-KM311 hhp2-K41R-FLAG*$_3$*:kanMX6 ade6-M210 ura4-D18 leu1-32 h*$^{-}$	This study
9124-2	*nda3-KM311 hhp2-K41R-4A-FLAG*$_3$*:kanMX6 ade6-M210 ura4-D18 leu1-32 h*$^{-}$	This study
5637	*nda3-KM311 ade6-M210 ura4-D18 leu1-32 h*$^{-}$	Lab stock
10439-2	*nda3-KM311 hhp2-4N-FLAG*$_3$*:kanMX6 ade6-M210 ura4-D18 leu1-32 h*$^{-}$	This study
10442-2	*nda3-KM311 hhp2-K41R-4N-FLAG*$_3$*:kanMX6 ade6-M210 ura4-D18 leu1-32 h*$^{-}$	This study
**Figure 3**		
100-2	*cdc2-asM17:bsd hhp2-FLAG*$_3$*:kanMX6 nda3-KM311 ade6-M210 ura4-D18 leu1-32 h*$^{?}$	This study
102-2	*cdc2-asM17:bsd hhp2-HA*$_3$*-TAP:kanMX6 nda3-KM311 ade6-M210 ura4-D18 leu1-32 h*$^{?}$	This study
5637	*nda3-KM311 ade6-M210 ura4-D18 leu1-32 h*$^{-}$	Lab stock
9121-2	*nda3-KM311 hhp2-K41R-FLAG*$_3$*:kanMX6 ade6-M210 ura4-D18 leu1-32 h*$^{+}$	This study

9119-2	*nda3-KM311 hhp2-FLAG$_3$:kanMX6 ade6-M210 ura4-D18 leu1-32 h$^+$*	This study
1373-2	*nda3-KM311 hhp2-3A-FLAG$_3$:kanMX6 ade6-M210 ura4-D18 leu1-32 h$^-$*	This study
3110-2	*nda3-KM311 hhp2-4A-FLAG$_3$:kanMX6 ade6-M210 ura4-D18 leu1-32 h$^-$*	This study
2311-2	*nda3-KM311 hhp2-7A-FLAG$_3$:kanMX6 ade6-M210 ura4-D18 leu1-32 h$^-$*	This study
**Figure 4**		
246	*ade6-M210 ura4-D18 leu1-32 h$^-$*	Lab stock
5637	*nda3-KM311 ade6-M210 ura4-D18 leu1-32 h$^-$*	Lab stock
2686-2	*nda3-KM311 hhp2-7A ade6-M210 ura4-D18 leu1-32 h$^-$*	This study
2688-2	*nda3-KM311 hhp2-K41R-7A ade6-M210 ura4-D18 leu1-32 h$^-$*	This study
19002	*rlc1-mNeonGreen:hygMX6 sid4-mNeonGreen:kanMX6 ade6-M21X ura4-D18 leu1-32 h$^-$*	Lab stock
3176-2	*rlc1-mNeonGreen:hygMX6 sid4-mNeonGreen:kanMX6 hhp2-7A ade6-M21X ura4-D18 leu1-32 h$^-$*	This study
3180-2	*rlc1-mNeonGreen:hygMX6 sid4-mNeonGreen:kanMX6 hhp2Δ::ura4$^+$ ade6-M21X ura4-D18 leu1-32 h$^-$*	This study
1193-2	*hhp2-7A ade6-M210 ura4-D18 leu1-32 h$^+$*	This study
16150-2	*plo1-ts35 ura4-D18 leu1-32 h$^-$*	Anderson et al., 2002
3473-3	*plo1-ts35 hhp2-7A ura4-D18 leu1-32 h$^-$*	This study
**Supplemental Figure 1**		
246	*ade6-M210 ura4-D18 leu1-32 h$^-$*	Lab stock
14041	*hhp1-FLAG$_3$:kanMX6 ura4-D18 leu1-32 ade6- M210 h$^-$*	Cullati et al., 2024
7549-2	*hhp1-K40R-FLAG$_3$:kanMX6 ura4-D18 leu1-32 ade6- M210 h$^-$*	Cullati et al., 2024
1721	*nda3-KM311 ade6-M210 ura4-D18 leu1-32 h$^-$*	Lab stock
14169	*nda3-KM311 hhp1-FLAG$_3$:kanMX6 ura4-D18 leu1-32 ade6- M210 h$^+$*	This study
7721-2	*nda3-KM311 hhp1-K40R-FLAG$_3$:kanMX6 ura4-D18 leu1-32 ade6-M21X h$^-$*	This study
**Supplemental Figure 3**		
247	*ade6-M210 ura4-D18 leu1-32 h$^+$*	Lab stock
721-2	*hhp2-3A ade6-M210 ura4-D18 leu1-32 h$^+$*	This study
8730-2	*hhp2-4A ade6-M210 ura4-D18 leu1-32 h$^+$*	This study
9221-2	*hhp2-4N ade6-M210 ura4-D18 leu1-32 h$^+$*	This study

1193-2	*hhp2-7A ade6-M210 ura4-D18 leu1-32* h^{+}	This study
19334	*hhp2-K41R ade6-M210 ura4-D18 leu1-32* h^{+}	This study
7683	*hhp2Δ::*$ura4^{+}$ *ade6-M210 ura4-D18 leu1-32* h^{+}	Bimbo et al., 2005
5637	*nda3-KM311 ade6-M210 ura4-D18 leu1-32* h^{-}	Lab stock
9121-2	*nda3-KM311 hhp2-K41R-*$FLAG_{3}$*:kanMX6 ade6-M210 ura4-D18 leu1-32* h^{+}	This study
10356-2	*nda3-KM311 hhp2-K41R,T345A-*$FLAG_{3}$*:kanMX6 ade6-M210 ura4-D18 leu1-32* h^{+}	This study
10358-2	*nda3-KM311 hhp2-K41R,S348A-*$FLAG_{3}$*:kanMX6 ade6-M210 ura4-D18 leu1-32* h^{-}	This study
10359-2	*nda3-KM311 hhp2-K41R-S350A,S351A-*$FLAG_{3}$*:kanMX6 ade6-M210 ura4-D18 leu1-32* h^{-}	This study
10360-2	*nda3-KM311 hhp2-K41R,S358A-*$FLAG_{3}$*:kanMX6 ade6-M210 ura4-D18 leu1-32* h^{-}	This study
10363-2	*nda3-KM311 hhp2-K41R,S380A,S381A-*$FLAG_{3}$*:kanMX6 ade6-M210 ura4-D18 leu1-32* h^{-}	This study
10364-2	*nda3-KM311 hhp2-K41R,S387A-*$FLAG_{3}$*:kanMX6 ade6-M210 ura4-D18 leu1-32* h^{-}	This study
10360-2	*nda3-KM311 hhp2-K41R,S358A-*$FLAG_{3}$*:kanMX6 ade6-M210 ura4-D18 leu1-32* h^{-}	This study
823-2	*nda3-KM311 hhp2-K41R,T345A,S358A-*$FLAG_{3}$*:kanMX6 ade6-M210 ura4-D18 leu1-32* h^{-}	This study
16831	*hhp2-mNeonGreen: kanMX6 ade6-M210 ura4-D18 leu1-32 h-*	Elmore et al., 2018
3319-2	*hhp2-3A-mNeonGreen:kanMX6 ade6-M210 ura4-D18 leu1-32* h^{+}	This study
3320-2	*hhp2-4A-mNeonGreen:kanMX6 ade6-M210 ura4-D18 leu1-32* h^{+}	This study
3321-2	*hhp2-7A-mNeonGreen:kanMX6 ade6-M210 ura4-D18 leu1-32* h^{+}	This study
**Supplemental Figure 4**		
7683	*hhp2Δ::*$ura4^{+}$ *ade6-M210 ura4-D18 leu1-32* h^{+}	Bimbo et al., 2005
19002	*rlc1-mNeonGreen:hygMX6 sid4-mNeonGreen:kanMX6 ade6-M21X ura4-D18 leu1-32* h^{-}	Lab stock
3176-2	*rlc1-mNeonGreen:hygMX6 sid4-mNeonGreen:kanMX6 hhp2-7A ade6-M21X ura4-D18 leu1-32* h^{-}	This study
3180-2	*rlc1-mNeonGreen:hygMX6 sid4-mNeonGreen:kanMX6 hhp2Δ::*$ura4^{+}$ *ade6-M21X ura4-D18 leu1-32* h^{-}	This study
246	*ade6-M210 ura4-D18 leu1-32* h^{-}	Lab stock
4040-2	*hhp1Δ::*$ura4^{+}$ *ade6-M210 ura4-D18 leu1-32* h^{+}	This study
16150-2	*plo1-ts35 ura4-D18 leu1-32* h^{-}	Anderson et al., 2002
3473-3	*plo1-ts35 hhp2-7A ura4-D18 leu1-32* h^{-}	This study

3477-3	*plo1-ts35 hhp2Δ::ura4$^+$ ura4-D18 leu1-32 h$^+$*	This study
3474-3	*plo1-ts35 hhp1Δ::ura4$^+$ ura4-D18 leu1-32 h$^+$*	This study
